# Identification and validation of single nucleotide polymorphic markers linked to Ug99 stem rust resistance in spring wheat

**DOI:** 10.1371/journal.pone.0171963

**Published:** 2017-02-27

**Authors:** Long-Xi Yu, Shiaoman Chao, Ravi P. Singh, Mark E. Sorrells

**Affiliations:** 1 United States Department of Agriculture–Agricultural Research Service, Plant Germplasm Introduction and Testing Research, Prosser, Washington, United States of America; 2 Plant Breeding and Genetics Section, School of Integrative Plant Science, Cornell University, Ithaca, New York, United States of America; 3 United States Department of Agriculture–Agricultural Research Service, Biosciences Research Laboratory, Fargo, North Dakota, United States of America; 4 International Maize and Wheat Improvement Center (CIMMYT), Apdo, El Batan, Mexico; Institute of Genetics and Developmental Biology Chinese Academy of Sciences, CHINA

## Abstract

Wheat stem rust (*Puccinia graminis* f. sp. *tritici* Eriks. and E. Henn.) is one of the most destructive diseases world-wide. Races belonging to Ug99 (or TTKSK) continue to cause crop losses in East Africa and threaten global wheat production. Developing and deploying wheat varieties with multiple race-specific genes or complex adult plant resistance is necessary to achieve durability. In the present study, we applied genome-wide association studies (GWAS) for identifying loci associated with the Ug99 stem rust resistance (SR) in a panel of wheat lines developed at the International Maize and Wheat Improvement Center (CIMMYT). Genotyping was carried out using the wheat 9K iSelect single nucleotide polymorphism (SNP) chip. Phenotyping was done in the field in Kenya by infection of *Puccinia graminis* f. sp. *tritici* race TTKST, the *Sr24*-virulent variant of Ug99. Marker-trait association identified 12 SNP markers significantly associated with resistance. Among them, 7 were mapped on five chromosomes. Markers located on chromosomes 4A and 4B overlapped with the location of the Ug99 resistance genes *SrND643* and *Sr37*, respectively. Markers identified on 7DL were collocated with *Sr25*. Additional significant markers were located in the regions where no *Sr* gene has been reported. The chromosome location for five of the SNP markers was unknown. A BLASTN search of the NCBI database using the flanking sequences of the SNPs associated with Ug99 resistance revealed that several markers were linked to plant disease resistance analogues, while others were linked to regulatory factors or metabolic enzymes. A KASP (Kompetitive Allele Specific PCR) assay was used for validating six marker loci linked to genes with resistance to Ug99. Of those, four co-segregated with the *Sr25*-pathotypes while the rest identified unknown resistance genes. With further investigation, these markers can be used for marker-assisted selection in breeding for Ug99 stem rust resistance in wheat.

## Introduction

Wheat stem rust, caused by a fungal pathogen *Puccinia graminis* f. sp. *tritici*, is one of the most destructive diseases of wheat. The deployment of stem rust resistance genes in modern wheat cultivars successfully controlled the disease until the new race of the pathogen named Ug99 (TTKSK, for North American nomenclature) was first reported in Uganda in 1999 [1]. Since then, the Ug99 race lineage has been observed in wheat fields in several countries in Africa and the Middle East. Due to the airborne transmission of disease, the Ug99 is expected to spread rapidly through these regions and further afield. The Ug99 pathogen is virulent against many resistance genes which have previously been applied in wheat against stem rust and can cause up to 100% crop losses [1–3]. Although efforts on developing Ug99-resistant varieties have been made, significant changes in the pathogen population and distribution are challenging as several variants have been identified within the Ug99 race lineage [2–4]. Developing new wheat varieties with diverse race-specific or durable resistance is the primary priority to mitigate the Ug99 threat worldwide.

To date, more than 60 stem rust resistance (*Sr*) genes have been identified and mapped to specific chromosome positions [5, 6]. However, only few of them are still effective against Ug99. Resistance to stem rust can be based on race-specific host pathogen recognition genes (R-genes) effective at all plant growth stages or multiple additive minor genes which confer adult plant resistance (APR). Durable and effective resistance to stem rust can be achieved by pyramiding multiple race-specific or slow rusting, minor resistance genes. The APR to stem rust in wheat is a complex trait and controlled by quantitative trait loci (QTL) that can provide more durable resistance better than a single, race-specific gene because of the race non-specificity of the resistance genes. To date, a total of five designated stem rust resistance genes conferring quantitative APR have been characterized. They are *Sr2*, *Sr55*, *Sr56*, *Sr57* and *Sr58*. Additional QTLs associated with wheat stem rust resistance have also been reported in diverse germplasm [7].

Pyramiding of more effective rust resistance genes into wheat cultivars with common background using rust bioassays is challenging due to the lack of isolates with specific avirulence/virulence gene combinations for assigning resistance genotypes. This is particularly true for broadly effective genes [8, 9]. Furthermore, field bioassays for the Ug99 lineage can only be conducted in regions where they are already present. High throughput diagnostic markers closely linked to stem rust resistance loci are needed to facilitate selection of desirable genotype combinations.

Although molecular markers have been used for marker-assisted selection (MAS) for wheat stem rust resistance, most of them are gel-based markers using fragment size comparison (http://maswheat.ucdavis.edu/protocols/stemrust/). It is time consuming and laborious. The Single Nucleotide Polymorphic (SNP) marker is the marker of choice for MAS because of their high abundance, widespread distribution throughout the genome, and their potential for high-throughput genotyping. In the present study, we first applied genome-wide association studies (GWAS) to identify SNP makers linked to Ug99 stem rust resistance loci in wheat lines developed at CIMMYT using the wheat 9K iSelect SNP chip. We then used a high throughput assay named Kompetitive Allele Specific PCR (KASP) for validating significant markers closely linked to the Ug99 resistance loci. Our goal was to develop a robust and high throughput platform that can be used in MAS for breeding wheat cultivars with improved resistance against Ug99 stem rust.

## Materials and methods

### Plant materials and bioassay for disease response

A total of 277 spring wheat lines from the 2nd and 5th Stem Rust Resistance Screening Nurseries (SRRSN) from CIMMYT were used for the field evaluation of stem rust resistance (S1 and S2 Tables). They were planted at the Kenya Agricultural Research Institute (KARI), Njoro during field seasons 2008, 2009 and 2010 for the 2nd SRRSN, and 2009 and 2010 for the 5th SRRSN with two replicates. The stem rust responses of the wheat lines were assessed in field plots as previously described [10]. Briefly, an artificial rust epidemic was created by infecting the spreaders using fresh urediniospores of *Puccinia graminis* f. sp. *tritici* race TTKST collected from field plots of a *Sr24* carrying spreader genotype planted at Njoro for rust increase. A suspension of freshly collected urediniospores in water was injected into individual plants (1–3 plants/m) within the border rows just prior to booting using a hypodermic syringe, on at least two occasions. Spreaders were also sprayed with urediniospore-mineral oil suspension at least twice during stem elongation. Stem rust was scored on the stem leaf sheath and true stem. Disease responses in the field were initially assessed at least twice between early to late dough stages when the susceptible control reached 80 to 100% infection and about a week later.

### SNP genotyping

DNA was extracted from young leaves of wheat lines using the CTAB protocol [11]. The wheat 9K iSelect SNP chip and the Infinium assay [12] was used for genotyping 189 wheat breeding lines from the 5^th^ SRRSN according to the manufacturer’s procedure (Illumina, San Diego, CA). The genotyping results were obtained using Illumina’s iScan instruments. Illumina’s GenomeStudio v2011.1 software was used for genotype calling and SNP clustering. To account for observed shifts in SNP clusters caused by differences in the number of duplicated gene copies detected during assays, a genotype calling algorithm was generated using an iterative procedure according to Cavanagh et al. [12].

### Marker-trait association

The corrected marker data and phenotypic data were used for marker-trait association. To control the possible population structure, a marker similarity matrix containing all lines (Kinship or K matrix) was generated using TASSEL v.5 [13]. Substructure within the wheat lines was also investigated using principal component analysis and the resulting covariance matrix (Q matrix) was used to correct the effect of population substructure. Both Q and K matrices were used in the mixed linear model (MLM) in TASSEL to correct for both population and family structure. A false discovery rate (FDR) of 0.05 was used as a threshold to identify significant markers associated with stem rust resistance [14].

### KASP assay

SNP markers associated with stem rust resistance identified by GWAS were validated using the KASP assay. The SNP contextual sequences were obtained from Dr. Eduard Akhunov (Kansas State University) and used for designing primers. For each marker, two allele-specific primers (one for each SNP allele) and one common (reverse) primer were designed for each KASP assay using a tool provided by LGC Genomics (www.lgcgenomics.com) based on the SNP locus sequence. Table 1 presents sequence information of six markers with two allele-specific primers and one common primer for each KASP assay. The KASP assays were designed by LGC Genomics and carried out according to the company’s protocol (http://lgcgenomics.com).

**Table 1:** Primer sequences of SNP markers for validation in wheat lines by KASP assay.

SNP	Allele1	Allele2	Allele-1 primer	Allele-2 primer	Common primer
JD_c6624_7769357 (7DL*)	A	G	CAAGAAGCCAATTGACCATGGCAAA	AAGAAGCCAATTGACCATGGCAAG	CTGATTGTTCACCAGCACTGGCATT
Ra_c25242_34807178 (7DL)	A	C	GGGATTCCTTCATCACAAGTTGGA	CCTTCATCACAAGTTGGC	GTTTCTCTTCTCTTGCCAAACCAGATTT
CAP7_c2912_1387634 (7DL*)	A	G	AACCGGTTACAAAGCCAAATCCAGA	CCGGTTACAAAGCCAAATCCAGG	ACTAGTGCTTGGTTTACCAATGTTCCTA
Ex_c5884_10325223 (7DL)	T	C	GTGCGCAGCTTACTTGCCAATATA	GCGCAGCTTACTTGCCAATATG	AATACCAGAATCGACAGATGTTGGAGTTT
Ex_c12556_19992307 (7BL)	A	G	ATTAGATCACGCTTTATCTGCTTGGAA	AGATCACGCTTTATCTGCTTGGAG	CTAGCACTRAGCTCGGCCTCAA
Ex_c2123_3988735 (7DS)	T	C	GTGGGGAGATAGGTGTGAAGGTA	GGGGAGATAGGTGTGAAGGTG	GTGTCACTTTTCGTTGGGAGTTACATTTT

Note

*, marker’s position was reassigned based on the linkage disequilibrium analysis.

## Results

### Population structure

**Fig 1:**
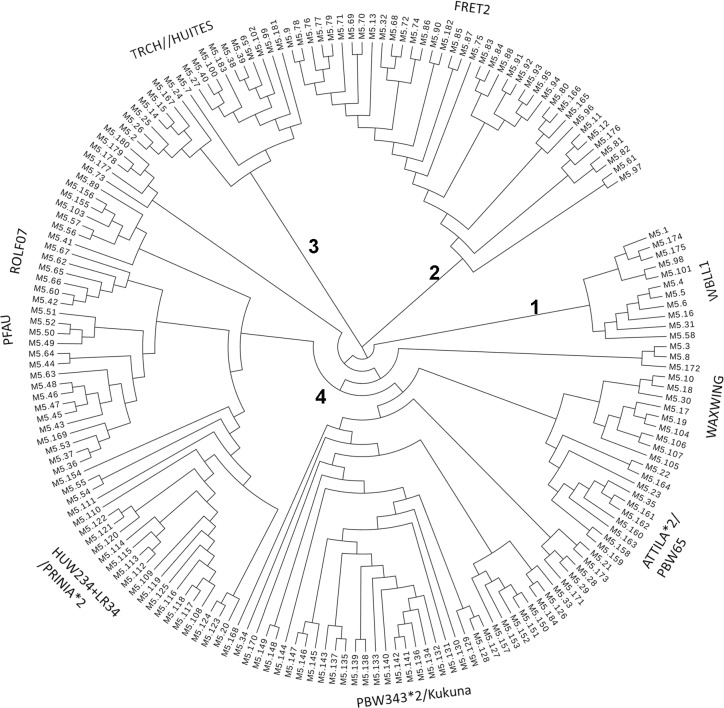
A dendrogram illustrating the clusters produced by hierarchical clustering of wheat lines in the 5^th^ SRRSN. Clusters with similar pedigrees and genetic backgrounds were named by their common parent.

To access the population structure, a cluster analysis was performed using the SNP data and four clusters were identified. A dendrogram was produced by hierarchical clustering (Fig 1). As diverse breeding lines were used in the GWAS, a wide-range of genetic background is expected in the association panel. Wheat lines with similar pedigrees or genetic backgrounds were clustered together. Cluster 1 contained wheat lines with the common parent “WBLL1”, cluster 2 contained wheat lines with the parent “FRET2” in common and cluster 3 contained wheat lines with the parent “TRCH” in the pedigree (Fig 1, upper). The 4^th^ cluster is a large cluster containing six subclusters with pedigrees containing “WAXWING”, “ATTILA*2/PBW65”, “PBW343*2/KUKUNA”, HUW234+LR34/PRINIA*2”, “PFAU” AND “ROLF07” (Fig 1, lower).

To confirm this finding, a principal component analysis (PCA) was performed using the same SNP data set and similar population structure was identified by the PCA (Data not shown). We therefore used the covariance matrix (PC1 and PC2) as Q-matrix together with K matrix in the mixed linear model for controlling the population structure effect in association mapping.

### Identification of SNP markers associated with Ug99 resistance

**Fig 2:**
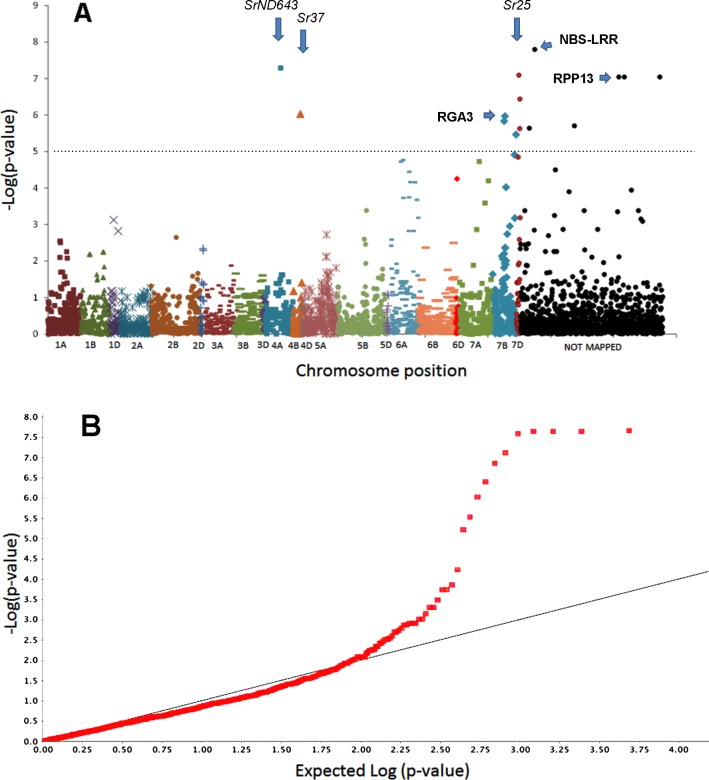
Manhattan and quantile-quantile (QQ) plots of association mapping of stem rust resistance in the 5^th^ SRRSN. A, each symbol represents a SNP. A false discovery rate of 0.05 was used for significant associations. Significant markers above the cutoff value of–log P = 5.0 (dotted line) were associated with SR resistance and they were listed in Table 2. The chromosome position was based on the 9k SNP linkage map. Each chromosome is numbered and distinguished by a color and shape of dots. The black dots represent unmapped markers. Arrows indicate the approximate position of known *Sr* genes and putative candidate genes for Ug99 resistance. B, The result of marker-trait association is illustrated in the QQ plot using observed against expected p-values (log transformed negatives).A mixed linear model controlled by Q and K matrices was used for analysis.

The resulting genotypic and phenotypic data were used for GWAS to identify markers associated with Ug99 resistance in the 5^th^ SRRSN (Fig 2A). The relative positions near the known *Sr* genes were indicated at the top of the respective chromosomes. Fig 2B illustrated the quantile-quantile plot (QQ) using observed against expected p-values. Using a cutoff FDR rate of 0.05, 12 significant markers were detected (Fig 2A above the dotted line, Table 2). Among them, seven were located on four chromosomes based on the consensus map constructed by Cavanagh et al. [12]. Marker Ex_c1373_2628597 on chromosomes 4A was located at the position of the previously reported stem resistance gene, *SrND643* [15]. Similarly, marker Ex_c30581_39482788 on chromosome 4B overlapped with the location of *Sr37* [7]. On chromosome 7B, one marker (Ex_c43096_49510164) was located where no Ug99-effective genes have been reported. Although *Sr17* was located on 7BS, it was reported to be ineffective to the Ug99 lineage [7]. Another marker Ex_c12556_19992307 was identified on 7BL in the same region where a QTL for stem rust resistance has been reported [16]. On 7D, a marker, Ex_c2123_3988735 was located on the short arm and coincided with the Ug99 resistance gene *SrTA100171[17]*. The other two, Ex_c5884_10325223 and Ra_c25242_34807178 were located on 7DL and overlapped with the location of *Sr25/Sr43*.

**Table 2:** Most significant SNP markers associated with stem rust resistance in the 5^th^ SRRSN wheat lines.

Trait	SNP ID	SNP name	Allele	Chr	Position	p-value	R^2^	Candidate
SR	1804	Ex_c1373_2628597	A/G	4A	73.0	7.76E-08	0.20	
SR	3287	Ex_c30581_39482788	T/C	4B	71.6	3.03E-06	0.15	GLSS
SR	3854	Ex_c43096_49510164	T/C	7B	65.6	4.10E-07	0.16	TIF4A
SR	1653	Ex_c12556_19992307	A/G	7B	163.9	9.65E-07	0.17	RGA3
SR	2592	Ex_c2123_3988735	A/G	7D	56.1	6.29E-06	0.14	IPT9
SR	4368	Ex_c5884_10325223	A/G	7D	139.7	1.41E-07	0.19	DSDS1
SR	7785	Ra_c25242_34807178	T/G	7D	141.2	2.25E-08	0.19	AG4
SR	6149	JD_c6624_7769357	A/G	NA	NA	2.34E-08	0.19	PP2C
SR	8369	RFL_Contig2671_2362005	T/C	NA	NA	2.34E-08	0.19	MDAR6
SR	5825	JD_c1314_1888758	A/G	NA	NA	2.34E-08	0.19	RPP13
SR	1073	CAP7_c2912_1387634	T/C	NA	NA	2.67E-08	0.19	NBS-LRR
SR	2175	Ex_c1690_3206784	A/G	NA	NA	6.00E-05	0.11	

Notes: Chr, chromosome. Known genes linked to SNPs by BLASTN in NCBI: GLS5, *Brachypodium distachyon* beta-galactosidase 5;, TIF4A, *Triticum aestivum* translation initiation factor 4A; RGA3, disease resistance gene analog RGA3; IPT9, *B*. *distachyon* importin-9; DSDS1, *B*. *distachyon* solanesyl-diphosphate synthase 1; AG4, *B*. *distachyon* enhancer of AG-4 protein 2; NBS-LRR, *H*. *vulgare* NBS-LRR resistance-like protein (RGH-2) gene; PP2C, *B*. *distachyon* protein phosphatase 2C; RPP13, putative disease resistance gene RPP13; MDAR6, *T*. *aestivum* MDAR6 gene for chloroplast protein product.

**Fig 3:**
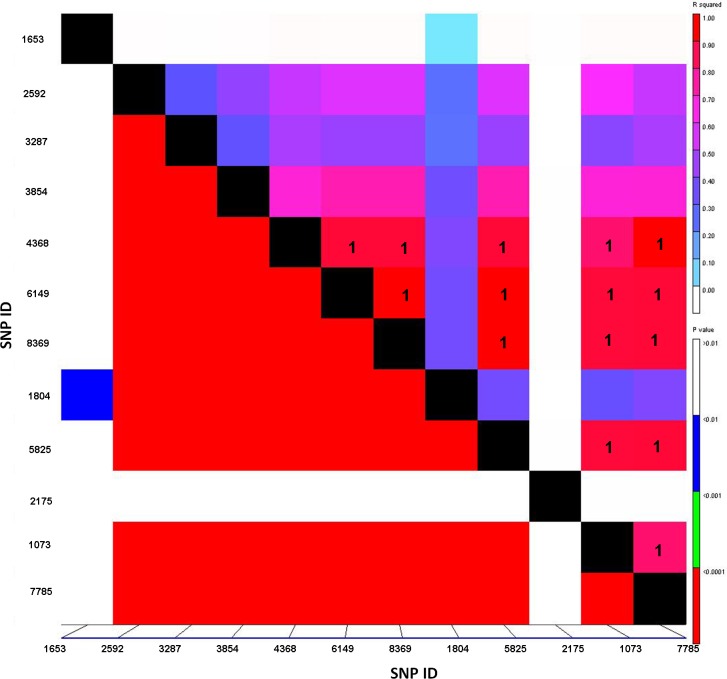
Linkage disequilibrium plots of significant SNP markers associated with stem rust resistance. R square (R^2^) and P-value of pair-wise analyses are indicated by color in the right-side bars. The SNP pairs with R^2^ value of “1” are indicated. The SNP IDs are presented on the X and Y axis. The detailed information for the markers is shown in Table 1.

Additional significant markers could not be mapped because their chromosomal locations are unknown (Table 2). We performed pair-wise analysis using linkage disequilibrium for all significant markers and the R^2^ and p-values are presented in Fig 3. The R^2^ value of “1” was obtained between four unmapped markers JD_c6624_7769357 (SNP ID 6149), RFL_Contig2671_2362005 (ID 8369), JD_c1314_1888758 (ID5825) and CAP7_c2912_1387634 (ID 1073) and two markers on 7DL Ex_c5884_10325223 (ID4368) and Ra_c25242_34807178 (7785) (Fig 3, labeled with “1”), suggesting that the four unmapped markers are linked to the loci on 7DL.Whereas marker Ex_c1690_3206784 (Fig 3, SNP ID 2175) showed no linkage with other significant markers.

### Assigning the loci associated with Ug99 resistance to putative candidate genes

To identify possible candidate genes linked to the resistance loci, a pairwise alignment using the BLASTN algorithm was performed using the flanking sequences of the significant markers against the NCBI (http://www.ncbi.nlm.nih.gov/) nucleotide acid databases. Three significant loci, Ex_c12556_19992307 on 7BL, CAP7_c2912_1387634 and JD_c1314_1888758 with unknown positions were linked to three plant disease resistance genes including a resistance gene analog RGA3, a member of the nucleotide-binding site (NBS)-leucine-rich repeat (LRR) gene, and a putative disease resistance gene, RPP13, respectively (Table 2). Another marker on 7BS (Ex_c43096_49510164) was linked to *T*.*aestivum* translation initiation factor 4A (TIF4A). Three loci on 7D, Ex_c2123_3988735, Ex_c5884_10325223 and Ra_c25242_34807178 were linked to importin-9 (IPT9), solanesyl-diphosphate synthase 1 (DSDS1) and enhancer of AG-4 protein 2 (AG4), respectively. Two unmapped markers, JD_c6624_7769357 and RFL_Contig2671_2362005 were linked to protein phosphatase 2C (PP2C) and a nuclear gene for chloroplast product (MDAR6), respectively (Table 2).

### Validation of SNP markers using KASP assay

**Fig 4:**
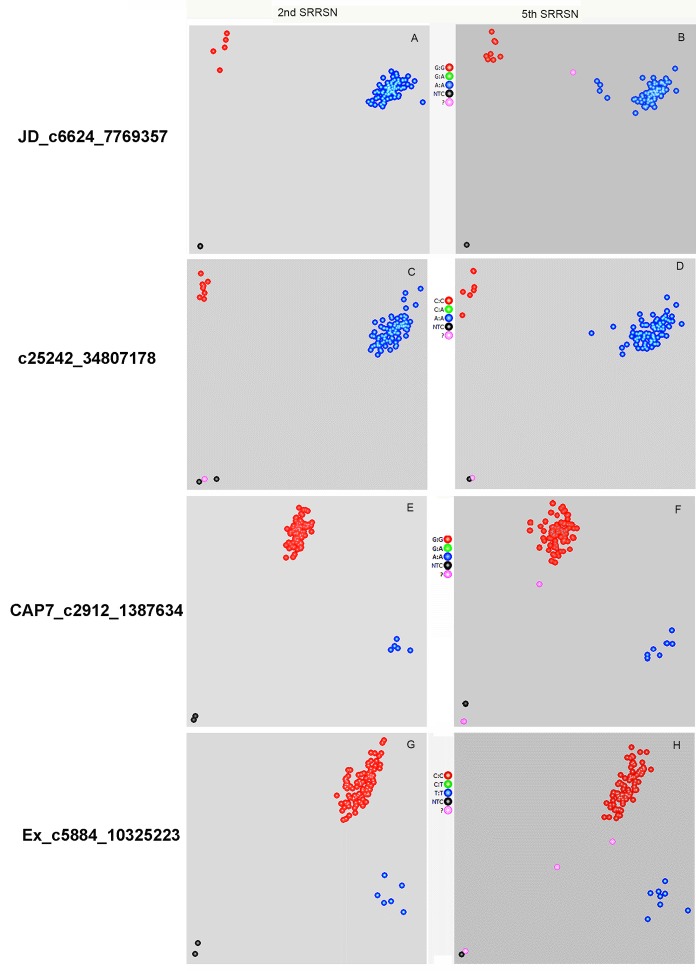
Discrimination plots of KASP assays of four SNPs linked to *Sr25* stem rust resistance locus. 2^nd^ SRRSN, the 2^nd^ Stem Rust Resistance Screen Nursery. 5^th^ SRRSN, the 5^th^ Stem Rust Resistance Screen Nursery (note: only the first 96-well plate samples are shown). Panels A, B, C and D, red dot = *Sr25*-allele; blue dot = non *Sr25*-allele. Panels E, F, G and H, blue dot = *Sr25*- allele; red dot = non *Sr25*-allele. For all panels, black dot = negative control, pink dot = undetermined.

To validate marker loci identified by GWAS, a KASP assay was used for genotyping. For cross validation, we expanded the population to 277 wheat lines included in two stem rust screening nurseries, the 2^nd^ and 5^th^ SRRSNs. We choose six most significant markers for the KASP assay (Table 1). For each SNP, three primers were designed and two alleles are labeled with either FAM or VIC for KASP assays. Their alleles and primer sequences are shown in Table 1. As a result, the blue or red colored dots represent the respective alleles in the allelic discrimination plots (Fig 4). For instance, marker c6624_7769357 identified five and 15 lines with genotype ‘G:G’ in the 2^nd^ and 5^th^ SRRSNs, respectively (Table 3, Fig 4A and 4B, red). The ‘G:G’ genotype detected wheat lines carrying *Sr25*, while the ‘A:A’ genotype represents wheat lines without *Sr25* (Fig 4A and 4B, blue). Marker Ra_c25242_34807178 identified eight and 15 lines carrying the ‘C:C’ genotype representing *Sr25* carriers in the 2^nd^ SRRN and 5^th^ SRRSN populations, respectively (Table 3, Fig 4C and 4D, red). Marker CAP7_c2912_1387634 identified five and 14 *Sr25* carriers with the ‘A:A’ genotype in the 2^nd^ and 5^th^ SRRSN populations, respectively (Table 3, Fig 4E and 4F, blue). The ‘G:G’ genotype represents non-*Sr25* carriers (Table 3, Fig 1E and 1F red). Interestingly, the “A:A” genotype was also detected in line M5_107 with adult plant resistance (ARP-R) without *Sr25*. Marker Ex_c5884_10325223 identified six and 16 lines that carry genotype ‘T:T’ in the 2^nd^ and 5^th^ SRRSN populations, respectively (Table 3, Fig 1G and 1H, blue). Most of these lines carry *Sr25*. The rest of lines without *Sr25* have genotype ‘C:C’ (Fig 1G and 1H, red). Among them, several lines had undetermined genotypes (Fig 1, pink). The rest two markers Ex_c2123_3988735 and Ex_c12556_19992307 were located on 7D and 7B, respectively. The former also identified some ‘C:C’ genotypes that was carried by *Sr25*-lines, however, genotype ‘T:T’ was also detected in two *Sr25*-lines. There was no significant correlation with Sr25 phenotype, although this marker is located on 7DS (56.1 cM) where an Ug99 resistance gene, *SrTA10171* was mapped [17]. Marker Ex_c12556_19992307 on 7BL showed no significant cosegregation with any known *Sr* phenotype in the germplasm tested. It is likely to identify a novel gene for Ug99 resistance.

**Table 3:** Validation of SNPs linked to stem rust resistance in the 2^nd^ and 5^th^ SRRSNs by KASP assay.

Line	Rust response	JD_c6624_7769357	CAP7_c2912_1387634	Ex_c5884_10325223	Ra_c25242_34807178	Ex_c2123_3988735	Ex_c12556_19992307
		(7DL*)	(7DL*)	(7DL)	(7DL)	(7DS)	(7BL)
H2_11	Sr25	G:G	A:A	T:T	C:C	C:C	A:A
H2_12	Sr25	G:G	A:A	T:T	C:C	C:C	A:A
H2_42	Sr25	G:G	A:A	T:T	C:C	C:C	G:A
H2_69	Sr25	G:G	A:A	T:T	C:C	C:C	A:A
H2_70	Sr25	G:G	A:A	T:T	C:C	C:C	G:A
M5_2	Sr25	G:G	A:A	T:T	C:C	T:T	G:A
M5_3	Sr25	G:G	A:A	T:T	C:C	C:C	G:A
M5_4	Sr25	G:G	A:A	T:T	C:C	C:C	G:A
M5_5	Sr25	G:G	A:A	T:T	C:C	C:C	G:A
M5_6	Sr25	G:G	A:A	T:T	C:C	C:C	G:A
M5_25	Sr25	G:G	A:A	T:T	C:C	C:C	A:A
M5_26	Sr25	G:G	A:A	T:T	C:C	C:C	G:A
M5_98	Sr25	G:G	A:A	T:T	C:C	C:C	A:A
M5_106	Sr25	G:G	A:A	T:T	C:C	C:C	G:A
M5_162	Sr25	G:G	A:A	T:T	C:C	C:C	A:A
M5_163	Sr25	G:G	A:A	T:T	C:C	T:T	A:A
M5_165	Sr25	G:G	A:A	T:T	C:C	C:C	G:A
M5_171	Sr25	G:G	A:A	T:T	C:C	C:C	G:A
M5_107	APR-R	G:G	A:A	T:T	C:C	C:C	G:A
M5_81	APR-R	G:G	?	T:T	C:C	T:T	G:A
H2_28	Sr-Sharp	A:A	G:G	T:T	C:C	T:T	G:G
H2_73	S	A:A	G:G	C:C	C:C	T:T	?
H2_30	-	A:A	G:G	C:C	A:A	T:T	G:G
H2_76	-	A:A	G:G	C:C	A:A	T:T	G:G
M5_57	APR-MR	A:A	G:G	C:C	A:A	T:T	G:G
M5_131	APR-MR	A:A	G:G	?	A:A	T:T	A:A
M5_10	APR-R	A:A	G:G	C:C	A:A	T:T	G:G
M5_11	APR-R	A:A	G:G	C:C	A:A	T:T	A:A
M5_12	APR-R	A:A	G:G	C:C	A:A	T:T	A:A
M5_22	APR-R	A:A	G:G	C:C	A:A	T:T	A:A
M5_33	APR-R	A:A	G:G	C:C	A:A	T:T	A:A
M5_36	APR-R	A:A	G:G	C:C	A:A	T:T	G:G
M5_37	APR-R	A:A	G:G	C:C	A:A	T:T	G:G
M5_41	APR-R	A:A	?	?	A:A	T:T	G:A
M5_45	APR-R	A:A	G:G	C:C	A:A	T:T	A:A
M5_46	APR-R	A:A	G:G	C:C	A:A	T:T	A:A
M5_47	APR-R	A:A	G:G	C:C	A:A	T:T	A:A
M5_48	APR-R	A:A	G:G	C:C	A:A	T:T	A:A
M5_49	APR-R	A:A	G:G	C:C	A:A	T:T	G:G
M5_50	APR-R	A:A	G:G	C:C	A:A	T:T	G:G
M5_52	APR-R	A:A	G:G	C:C	A:A	T:T	G:G
M5_53	APR-R	A:A	G:G	C:C	A:A	T:T	G:G
M5_66	APR-R	A:A	G:G	C:C	A:A	T:T	A:A
M5_68	APR-R	A:A	G:G	C:C	A:A	T:T	G:G
M5_69	APR-R	A:A	G:G	C:C	A:A	T:T	A:A
M5_72	APR-R	A:A	G:G	C:C	A:A	T:T	G:G
M5_83	APR-R	A:A	G:G	C:C	A:A	T:T	A:A
M5_85	APR-R	A:A	G:G	C:C	A:A	T:T	G:G
M5_99	APR-R	A:A	G:G	C:C	A:A	T:T	G:G
M5_102	APR-R	A:A	G:G	C:C	A:A	T:T	G:G
M5_126	APR-R	A:A	G:G	C:C	A:A	T:T	A:A
M5_130	APR-R	A:A	G:G	C:C	A:A	T:T	A:A
M5_137	APR-R	A:A	G:G	C:C	A:A	T:T	A:A
M5_138	APR-R	A:A	G:G	C:C	A:A	T:T	A:A
M5_142	APR-R	A:A	G:G	C:C	A:A	T:T	A:A
M5_143	APR-R	A:A	G:G	C:C	A:A	T:T	A:A
M5_147	APR-R	A:A	G:G	C:C	A:A	T:T	A:A
M5_148	APR-R	A:A	G:G	C:C	A:A	T:T	A:A
M5_149	APR-R	A:A	G:G	C:C	A:A	T:T	A:A
M5_154	APR-R	A:A	G:G	C:C	A:A	T:T	A:A
M5_164	APR-R	A:A	G:G	C:C	A:A	T:T	A:A
M5_172	APR-R	A:A	G:G	C:C	A:A	T:T	G:G
M5_173	APR-R	A:A	G:G	C:C	A:A	T:T	A:A
M5_182	APR-R	A:A	G:G	C:C	A:A	T:T	A:A
M5_7	APR-RMR	A:A	G:G	C:C	A:A	?	A:A
M5_9	APR-RMR	A:A	G:G	C:C	A:A	T:T	G:G
M5_13	APR-RMR	A:A	G:G	C:C	A:A	T:T	A:A
M5_14	APR-RMR	A:A	G:G	C:C	A:A	T:T	G:G
M5_15	APR-RMR	A:A	G:G	C:C	A:A	T:T	G:G
M5_17	APR-RMR	A:A	G:G	C:C	A:A	T:T	A:A
M5_18	APR-RMR	A:A	G:G	C:C	A:A	T:T	G:G
M5_19	APR-RMR	A:A	?	?	A:A	?	?
M5_21	APR-RMR	A:A	G:G	C:C	A:A	T:T	A:A
M5_23	APR-RMR	A:A	G:G	C:C	A:A	T:T	A:A
M5_24	APR-RMR	A:A	G:G	C:C	A:A	T:T	A:A
M5_30	APR-RMR	A:A	G:G	C:C	A:A	T:T	?
M5_31	APR-RMR	A:A	G:G	C:C	A:A	T:T	A:A
M5_32	APR-RMR	A:A	G:G	C:C	A:A	T:T	A:A
M5_34	APR-RMR	A:A	G:G	C:C	A:A	T:T	G:G
M5_35	APR-RMR	A:A	G:G	C:C	A:A	T:T	A:A
M5_38	APR-RMR	A:A	G:G	C:C	A:A	T:T	A:A
M5_39	APR-RMR	A:A	G:G	C:C	A:A	T:T	A:A
M5_40	APR-RMR	A:A	G:G	C:C	A:A	T:T	G:G
M5_43	APR-RMR	A:A	G:G	C:C	A:A	T:T	A:A
M5_44	APR-RMR	A:A	G:G	C:C	A:A	T:T	G:G
M5_51	APR-RMR	A:A	G:G	C:C	A:A	T:T	G:G
M5_54	APR-RMR	A:A	G:G	C:C	A:A	T:T	G:G
M5_58	APR-RMR	A:A	G:G	C:C	A:A	T:T	A:A
M5_59	APR-RMR	A:A	G:G	C:C	A:A	T:T	G:G
M5_60	APR-RMR	A:A	G:G	C:C	A:A	T:T	A:A
M5_61	APR-RMR	A:A	G:G	C:C	A:A	T:T	A:A
M5_62	APR-RMR	A:A	G:G	C:C	A:A	T:T	A:A
M5_64	APR-RMR	A:A	G:G	C:C	A:A	T:T	A:A
M5_65	APR-RMR	A:A	G:G	C:C	A:A	T:T	A:A
M5_67	APR-RMR	A:A	G:G	C:C	A:A	T:T	A:A
M5_70	APR-RMR	A:A	G:G	C:C	A:A	T:T	A:A
M5_71	APR-RMR	A:A	G:G	C:C	A:A	T:T	A:A
M5_73	APR-RMR	A:A	G:G	C:C	A:A	T:T	A:A
M5_74	APR-RMR	A:A	G:G	C:C	A:A	T:T	G:G
M5_75	APR-RMR	A:A	G:G	C:C	A:A	T:T	G:G
M5_76	APR-RMR	A:A	G:G	C:C	A:A	T:T	G:A
M5_77	APR-RMR	A:A	G:G	C:C	A:A	T:T	A:A
M5_78	APR-RMR	A:A	G:G	C:C	A:A	T:T	A:A
M5_79	APR-RMR	A:A	G:G	C:C	A:A	T:T	?
M5_80	APR-RMR	A:A	G:G	C:C	A:A	T:T	G:G
M5_82	APR-RMR	A:A	G:G	C:C	A:A	T:T	G:G
M5_84	APR-RMR	A:A	G:G	C:C	A:A	T:T	A:A
M5_86	APR-RMR	A:A	G:G	C:C	A:A	T:T	G:G
M5_87	APR-RMR	A:A	G:G	C:C	A:A	T:T	G:G
M5_88	APR-RMR	A:A	G:G	C:C	A:A	?	G:G
M5_90	APR-RMR	A:A	G:G	C:C	A:A	T:T	A:A
M5_92	APR-RMR	A:A	G:G	C:C	A:A	T:T	G:G
M5_93	APR-RMR	A:A	G:G	C:C	A:A	T:T	G:G
M5_94	APR-RMR	A:A	G:G	C:C	A:A	T:T	G:G
M5_95	APR-RMR	A:A	G:G	C:C	A:A	T:T	G:G
M5_96	APR-RMR	A:A	G:G	C:C	A:A	T:T	A:A
M5_97	APR-RMR	A:A	G:G	C:C	A:A	T:T	G:G
M5_100	APR-RMR	A:A	G:G	C:C	A:A	T:T	G:G
M5_104	APR-RMR	A:A	G:G	C:C	A:A	T:T	G:G
M5_105	APR-RMR	A:A	G:G	C:C	A:A	T:T	G:G
M5_127	APR-RMR	A:A	G:G	C:C	A:A	T:T	G:A
M5_128	APR-RMR	A:A	G:G	C:C	A:A	T:T	A:A
M5_132	APR-RMR	A:A	G:G	C:C	A:A	T:T	A:A
M5_133	APR-RMR	A:A	G:G	C:C	A:A	T:T	A:A
M5_134	APR-RMR	A:A	G:G	C:C	A:A	T:T	A:A
M5_135	APR-RMR	A:A	G:G	C:C	A:A	T:T	G:G
M5_136	APR-RMR	A:A	G:G	C:C	A:A	T:T	A:A
M5_139	APR-RMR	A:A	G:G	C:C	A:A	T:T	A:A
M5_140	APR-RMR	A:A	G:G	C:C	A:A	T:T	A:A
M5_141	APR-RMR	A:A	G:G	C:C	A:A	T:T	A:A
M5_144	APR-RMR	A:A	G:G	C:C	A:A	T:T	G:G
M5_145	APR-RMR	A:A	G:G	C:C	A:A	T:T	A:A
M5_146	APR-RMR	A:A	G:G	C:C	A:A	T:T	A:A
M5_150	APR-RMR	A:A	G:G	C:C	A:A	T:T	?
M5_151	APR-RMR	A:A	G:G	C:C	A:A	T:T	A:A
M5_152	APR-RMR	A:A	G:G	C:C	A:A	T:T	A:A
M5_153	APR-RMR	A:A	G:G	C:C	A:A	T:T	G:G
M5_158	APR-RMR	A:A	G:G	C:C	A:A	T:T	A:A
M5_166	APR-RMR	A:A	G:G	C:C	A:A	T:T	A:A
M5_167	APR-RMR	A:A	G:G	C:C	A:A	T:T	G:G
M5_168	APR-RMR	A:A	G:G	C:C	A:A	T:T	G:G
M5_169	APR-RMR	A:A	G:G	C:C	A:A	T:T	A:A
M5_170	APR-RMR	A:A	G:G	C:C	A:A	T:T	A:A
M5_176	APR-RMR	A:A	G:G	C:C	A:A	T:T	A:A
M5_177	APR-RMR	A:A	G:G	C:C	A:A	T:T	A:A
M5_178	APR-RMR	A:A	G:G	C:C	A:A	T:T	A:A
M5_179	APR-RMR	A:A	G:G	C:C	A:A	T:T	G:G
M5_180	APR-RMR	?	G:G	C:C	A:A	T:T	A:A
M5_181	APR-RMR	A:A	G:G	C:C	A:A	T:T	G:G
H2_2	MR	A:A	G:G	C:C	A:A	T:T	A:A
H2_13	MR	A:A	G:G	C:C	A:A	T:T	A:A
H2_14	MR	A:A	G:G	C:C	A:A	T:T	A:A
H2_17	MR	A:A	G:G	C:C	A:A	T:T	G:G
H2_25	MR	A:A	G:G	C:C	A:A	T:T	G:G
H2_26	MR	A:A	G:G	C:C	A:A	T:T	A:A
H2_31	MR	A:A	G:G	C:C	A:A	T:T	G:G
H2_33	MR	A:A	G:G	C:C	A:A	T:T	G:G
H2_34	MR	A:A	G:G	C:C	A:A	T:T	G:G
H2_38	MR	A:A	G:G	C:C	A:A	T:T	G:G
H2_40	MR	A:A	G:G	C:C	A:A	T:T	G:G
H2_47	MR	A:A	G:G	C:C	A:A	T:T	G:G
H2_52	MR	A:A	G:G	C:C	A:A	T:T	A:A
H2_60	MR	A:A	G:G	C:C	A:A	T:T	A:A
H2_65	MR	A:A	G:G	C:C	A:A	T:T	A:A
H2_68	MR	A:A	G:G	C:C	A:A	T:T	A:A
H2_77	MR	A:A	G:G	C:C	A:A	T:T	A:A
H2_78	MR	A:A	G:G	C:C	A:A	T:T	G:G
H2_80	MR	A:A	G:G	C:C	A:A	T:T	G:G
H2_81	MR	A:A	G:G	C:C	A:A	T:T	A:A
H2_84	MR	A:A	G:G	C:C	A:A	T:T	G:G
H2_85	MR	A:A	G:G	C:C	A:A	T:T	A:A
H2_86	MR	A:A	G:G	C:C	A:A	T:T	A:A
H2_93	MR	A:A	G:G	C:C	A:A	T:T	A:A
H2_41	MR-MS	A:A	G:G	C:C	A:A	T:T	A:A
H2_43	MR-MS	A:A	G:G	C:C	A:A	T:T	A:A
H2_44	MR-MS	A:A	G:G	C:C	A:A	T:T	G:G
H2_45	MR-MS	A:A	G:G	C:C	A:A	T:T	A:A
H2_46	MR-MS	A:A	G:G	C:C	A:A	T:T	A:A
H2_49	MR-MS	A:A	G:G	C:C	A:A	T:T	G:G
H2_50	MR-MS	A:A	G:G	C:C	?	T:T	G:G
H2_51	MR-MS	A:A	G:G	C:C	A:A	T:T	G:G
H2_55	MR-MS	A:A	G:G	C:C	A:A	T:T	G:G
H2_56	MR-MS	A:A	G:G	C:C	A:A	T:T	G:G
H2_57	MR-MS	A:A	G:G	C:C	A:A	T:T	G:G
H2_58	MR-MS	A:A	G:G	C:C	A:A	T:T	A:A
H2_59	MR-MS	A:A	G:G	C:C	A:A	T:T	G:G
H2_62	MR-MS	A:A	G:G	C:C	A:A	T:T	A:A
H2_1	MS	A:A	G:G	C:C	A:A	T:T	A:A
H2_3	MS	A:A	G:G	C:C	A:A	T:T	A:A
H2_10	MS	A:A	G:G	C:C	A:A	T:T	A:A
H2_20	MS	A:A	G:G	C:C	A:A	T:T	G:G
H2_21	MS	A:A	G:G	C:C	A:A	T:T	G:G
H2_22	MS	A:A	G:G	C:C	A:A	T:T	A:A
H2_27	MS	A:A	G:G	C:C	A:A	T:T	G:G
H2_29	MS	A:A	G:G	C:C	A:A	?	G:G
H2_36	MS	A:A	G:G	C:C	A:A	T:T	G:G
H2_37	MS	A:A	G:G	C:C	A:A	T:T	G:G
H2_39	MS	A:A	G:G	C:C	A:A	T:T	A:A
H2_48	MS	A:A	G:G	C:C	A:A	T:T	A:A
H2_54	MS	A:A	G:G	C:C	A:A	T:T	A:A
H2_61	MS	A:A	G:G	C:C	A:A	T:T	G:G
H2_66	MS	A:A	G:G	C:C	A:A	T:T	A:A
H2_74	MS	A:A	G:G	C:C	A:A	T:T	G:G
H2_89	MS	A:A	G:G	C:C	A:A	T:T	G:G
M5_184	MS-S	A:A	G:G	C:C	A:A	T:T	G:G
H2_87	R	A:A	G:G	C:C	A:A	T:T	G:G
H2_5	R-MR	A:A	G:G	C:C	A:A	T:T	A:A
H2_6	R-MR	A:A	G:G	C:C	A:A	T:T	G:G
H2_8	R-MR	A:A	G:G	C:C	A:A	T:T	A:A
H2_16	R-MR	A:A	G:G	C:C	A:A	T:T	A:A
H2_24	R-MR	A:A	G:G	C:C	A:A	T:T	G:G
H2_35	R-MR	A:A	G:G	C:C	A:A	T:T	A:A
H2_53	R-MR	A:A	G:G	C:C	A:A	T:T	A:A
H2_63	R-MR	A:A	G:G	C:C	A:A	T:T	A:A
H2_79	R-MR	A:A	G:G	C:C	A:A	T:T	G:A
H2_82	R-MR	A:A	G:G	C:C	A:A	T:T	A:A
H2_83	R-MR	A:A	G:G	C:C	A:A	T:T	A:A
H2_90	R-MR	A:A	G:G	C:C	A:A	T:T	?
H2_91	R-MR	A:A	G:G	C:C	A:A	T:T	G:G
H2_92	R-MR	A:A	G:G	C:C	A:A	T:T	A:A
H2_9	S	A:A	G:G	C:C	A:A	T:T	G:G
H2_18	S	A:A	G:G	C:C	A:A	T:T	G:G
H2_19	S	A:A	G:G	C:C	A:A	T:T	G:G
H2_32	S	A:A	G:G	C:C	A:A	T:T	G:G
H2_64	S	A:A	G:G	C:C	A:A	T:T	A:A
H2_75	S	A:A	G:G	C:C	C:C	T:T	G:G
M5_91	APR-RMR	A:A	G:G	C:C	A:A	T:T	G:G
M5_63	Sr26	A:A	G:G	C:C	A:A	T:T	A:A
M5_89	Sr26	A:A	G:G	C:C	A:A	T:T	A:A
M5_103	Sr26	A:A	G:G	C:C	A:A	?	A:A
M5_129	Sr26	A:A	G:G	C:C	A:A	T:T	A:A
M5_155	Sr26	A:A	G:G	C:C	A:A	T:T	A:A
M5_156	Sr26	A:A	G:G	C:C	A:A	T:T	A:A
M5_157	Sr26	A:A	G:G	C:C	A:A	T:T	G:G
M5_160	Sr26	A:A	G:G	C:C	A:A	T:T	A:A
M5_161	Sr26	A:A	G:G	C:C	A:A	T:T	A:A
M5_42	Sr26?	A:A	G:G	C:C	A:A	T:T	A:A
M5_159	Sr26?	A:A	G:G	C:C	A:A	T:T	A:A
H2_15	Sr33?	A:A	G:G	C:C	A:A	T:T	G:G
H2_67	SrHUW234	A:A	G:G	C:C	A:A	T:T	A:A
M5_20	SrHuw234	A:A	G:G	C:C	A:A	T:T	A:A
M5_55	SrHuw234	A:A	G:G	C:C	A:A	T:T	G:G
M5_56	SrHuw234	A:A	G:G	C:C	A:A	T:T	G:G
M5_109	SrHuw234	A:A	G:G	C:C	A:A	T:T	A:A
M5_110	SrHuw234	A:A	G:G	C:C	A:A	T:T	A:A
M5_111	SrHuw234	A:A	G:G	C:C	A:A	T:T	G:G
M5_112	SrHuw234	A:A	G:G	C:C	A:A	T:T	A:A
M5_116	SrHuw234	A:A	G:G	C:C	A:A	T:T	A:A
M5_117	SrHuw234	A:A	G:G	C:C	A:A	T:T	A:A
M5_118	SrHuw234	A:A	G:G	C:C	A:A	T:T	A:A
M5_119	SrHuw234	A:A	G:G	C:C	A:A	T:T	A:A
M5_120	SrHuw234	A:A	G:G	C:C	A:A	T:T	?
M5_121	SrHuw234	A:A	G:G	C:C	A:A	T:T	A:A
M5_122	SrHuw234	A:A	G:G	C:C	A:A	T:T	A:A
M5_123	SrHuw234	A:A	G:G	C:C	A:A	T:T	A:A
M5_124	SrHuw234	A:A	G:G	C:C	A:A	T:T	A:A
M5_125	SrHuw234	A:A	G:G	C:C	A:A	T:T	A:A
H2_7	SrHUW234	A:A	G:G	C:C	A:A	T:T	A:A
M5_108	SrHuw234/Sr26	A:A	G:G	C:C	A:A	T:T	A:A
M5_113	SrHuw234/Sr26	A:A	G:G	C:C	A:A	T:T	A:A
M5_114	SrHuw234/Sr26	A:A	G:G	C:C	A:A	T:T	G:G
M5_115	SrHuw234/Sr26	A:A	G:G	C:C	A:A	T:T	A:A
H2_4	Sr-ND643	A:A	G:G	C:C	A:A	T:T	A:A
H2_71	SrSha7	A:A	G:G	C:C	A:A	T:T	G:G
H2_72	SrSha7	A:A	G:G	C:C	A:A	T:T	G:G
H2_94	SrSha7	A:A	G:G	C:C	A:A	T:T	A:A
M5_8	SrSha7	A:A	G:G	C:C	A:A	T:T	G:G
M5_27	SrSha7	A:A	G:G	C:C	A:A	T:T	G:G
M5_28	SrSha7	A:A	G:G	C:C	A:A	T:T	G:G
M5_29	SrSha7	A:A	G:G	C:C	A:A	T:T	G:G
M5_183	SrSha7	A:A	G:G	C:C	A:A	T:T	A:A
H2_88	SrSynthetic	A:A	G:G	C:C	A:A	T:T	G:G
H2_23	SrTmp	A:A	G:G	C:C	A:A	T:T	G:G
M5_1	SrTmp	A:A	G:G	C:C	A:A	T:T	G:G
M5_101	SrTnmu	A:A	G:G	C:C	A:A	T:T	A:A
M5_174	SrTnmu	A:A	G:G	C:C	A:A	T:T	G:G
M5_175	SrTnmu	A:A	G:G	C:C	A:A	T:T	G:G

Notes: H2 = the 2^nd^ SRRSN. M5 = the 5^th^ SRRSN. SR, plant response to the stem rust pathogens. APR, adult plant resistance. R = resistance. S = susceptible. MR = moderate resistance. Yellow = *Sr25* haplotypes. Other colors = non-*Sr25* haplotypes.

*, marker’s position was reassigned based on the linkage disequilibrium analysis.?, result could not be determined.

## Discussion

### Marker loci associated with Ug99 resistance

Of markers identified in the present study, 10 were mapped and they were located on two homoeologous groups (Groups 4 and 7) based on the 9K consensus map [12]. On Group 4, marker Ex_c1373_2628597 located on 4A is in the same region where the temporarily designated gene, *SrND643*, was mapped [15]. This gene was identified from wheat breeding line ‘ND643/2*Webill1 (or WBLL1)’, and is effective against the Ug99 group races at both seedling and adult growth stages. “WBLL1” was also present based on pedigree in a number of wheat lines used in this study. Therefore, the marker Ex_c1373_2628597 is likely to identify the same gene as *SrND643*. Marker Ex_c30581_39482788 on 4B shared the same chromosome location with *Sr37*, a gene that is effective against the Ug99 lineage. *Sr37* was originally transferred from wild *Triticum timopheevii var*. *araraticum* [5] but was not successfully utilized in wheat breeding. Of significant markers on 7B, marker Ex_c43096_49510164 located at the position (65 cM) where no Ug99 resistance gene has been reported, although *Sr17* was mapped in the same region, it is ineffective against Ug99 [7]. On the other hand, marker Ex_c12556_19992307 identified on 7BL 163.9 cM) overlapped with the location of the stem rust resistance QTL reported by Bansal et al. [16]. Moreover, we identified a DArT marker associated with stem rust resistance in the same region in our previous study [10]. The consistent identification of loci associated with stem rust resistance in this region suggests that there is likely a novel *Sr* gene on 7BL contributing to Ug99 resistance.

Additional markers with unknown chromosomal positions were also identified with Ug99 resistance. For instance, based on the LD analysis, among five unknowns, four markers, JD_c6624_7769357, RFL_Contig2671_2362005, JD_c1314_1888758 and CAP7_c2912_1387634 were tightly linked to the *Sr25* locus, a major effective gene against the Ug99 lineage (Fig 3). Two of them (JD_c6624_7769357 and CAP7_c2912_1387634) were later confirmed by the KASP assay where 90 and 95% cosegregation with *Sr25* phenotypes were obtained, respectively (Table 4).

**Table 4:** Validation of SNP markers linked to Sr25 in wheat breeding lines with known gene resources.

SNP marker	*Sr25* allele	Other allele	No. of *Sr25* genotype	No. of *Sr25* phenotype	Accuracy %
JD_c6624_7769357	G:G	A:A	20	18	90
Ra_c25242_34807178	C:C	A:A	23	18	78
CAP7_c2912_1387634	A:A	G:G	19	18	95
Ex_c5884_10325223	T:T	C:C	21	18	86

### Markers associated with Ug99 resistance linked to known disease resistance genes

Among the resistance loci identified in the present study, three markers (Ex_c12556_19992307, CAP7_c2912_1387634 and JD_c1314_1888758) were linked to three disease resistance genes (RGA3, NBS-LRR and RPP13). The RGA13 is a member of resistance gene analogs in plants that triggers a defense system to restrict the pathogen growth. The RGA3 was linked to marker Ex_c12556_19992307 which is located on 7BL where a QTL and a DArT marker associated with stem rust resistance has been reported [10, 16]. The RGA3 may be a putative candidate underlying the QTL interval. The NBS-LRR genes play roles in disease resistance in plants through similar mechanisms as other RGAs [18]. The identification of the NBS-LRR linked to the marker CAP7_c2912_1387634 at the *Sr25* locus suggests that the NBS-LRR is a putative candidate underlying *Sr25*. Furthermore, at the same locus, marker JD_c1314_1888758 was linked to RPP13, another member of the NBS-LRR gene family. It has been reported that RPP13 contributes plant resistance to downy mildew in Arabidopsis [19]. Therefore, RPP13 may be another candidate for *Sr25* and play a role in plant resistance to Ug99.

### Marker loci linked to *Sr25*

Gene *Sr25* is one the few race-specific genes effective against all races belonging to the Ug99 lineage [8, 9]. *Sr25* was transferred into wheat in a translocation on 7DL from *Thinopyrum* (*Th*) *ponticum (Podp*.*)* by Barkworth and Dewey [20]. However, the use of germplasm containing *Sr25* was limited due to the linkage of *Sr25* with another *Th*. *ponticum* derived gene resulting in undesirable yellow flour. Later on, mutant lines, Agatha-28 and Agatha-235, with reduced levels of yellow pigment in flour were produced [21]. One of the mutant lines containing *Sr25* was backcrossed into the Australian wheat backgrounds and has been used in the CIMMYT breeding program where it is present in the variety ‘Wheatear’ [22].

Diagnostic markers for *Sr25* reported in this study can facilitate MAS for Ug99 resistance in wheat since *Sr25* is effective against races of the Ug99 lineage [1, 8, 23]. In the present study, four SNP markers were found to be closely linked to *Sr25* and were able to predict wheat lines carrying *Sr25* with marker CAP7_c2912_1387634 having the highest accuracy (95%) (Table 4). The other two markers, Ex_c5884_10325223 and JD_c6624_7769357 were able to detect *Sr25*-cariers at 86 and 90% accuracies, respectively (Table 4), while marker Ra_c25242_34807178 had the lowest accuracy (78%).

Our results show that wheat lines that possess the *Sr25* resistance locus fall into the haplotype of ‘T-C-G-A’ in the 2^nd^ and 5^th^ SRRSN populations with the marker combination of Ex_c5884_10325223- Ra-c25242_34807178-JD_c6624_7769357- CAP7_c2912_1387634 (Table 3, yellow highlighted). The *Sr25*-haplotype detected all *Sr25*-lines in 277 wheat lines tested in the present study. Most of the *Sr25*-lines with the ‘T-C-G-A’ haplotype had ‘Wheatear’ parent in their pedigrees, which is consistent with the introduction of gene *Sr25* in this variety. Although a similar haplotype was found in line M5_107, *This line* neither responded to *Sr25* nor was ‘Wheatear’ in the pedigree. However, M5_107 showed “APR-R” response against Ug99 (S1 and S2 Tables). A possible explanation is that *Sr25* allele might be accidently introduced to this line or a mistake in phenotyping. However, the use of haplotypes with the combination of four markers increased the diagnostic accuracy.

Among the 277 lines screened with markers reported in this study, only 18 lines carried the *Sr25*-genotype. This is in agreement with the limited use of this resistance gene in breeding programs [5]. However, we anticipate the use of *Sr25* will increase since increasing efforts have been made to develop cultivars with stem rust resistance due to the threat posed by races of the Ug99 lineage. Gene *Sr25* is among a few race-specific genes effective against these races [8, 9]. Furthermore, *Sr25* without yellow flour has been recently transferred into Australian and CIMMYT wheat backgrounds [21], and there is evidence to suggest that the *Th*. *ponticum* segment carrying *Sr25* can increase yield potential under irrigated conditions [24, 25].

## Conclusion

In the present study, we applied genome-wide association studies to identify SNP makers linked to Ug99 stem rust resistance loci using the 9K SNP chip. Marker-trait association analysis identified 12 SNPs significantly associated with the stem rust resistance. They were located on 4 chromosomes (4A, 4B, 7B and 7D). Markers located on 4A, 4B and 7D were overlapped with the reported genes SrND643, Sr37 and Sr25, respectively. Whereas, markers identified on 7B were located in the regions where no *Sr* gene has been reported, although a QTL has been reported in the same region as the marker Ex_c12556_19992307 identified in the present study. Several markers associated with stem rust resistance were linked to putative candidate genes that play roles in plant disease resistance. Six markers linked to the resistance were validated in 277 breeding lines using a high-throughput KASP assay. The result indicated that four of them cosegregated with *Sr25* genotypes while the other two are likely to be linked to other genes. The diagnostic ability of these markers and the high throughput platform characterized in the present study may be used for marker-assisted selection, especially for *Sr25*, and would be beneficial for accelerating breeding programs to improve wheat resistance to stem rust such as Ug99.

## Supporting information

S1 Table Genotyping results of markers linked to *Sr25*, stem rust responses and pedigrees of wheat lines in the 2^nd^ and 5^th^ stem rust resistance screening nurseries. (XLSX)

S2 Table Genotyping results of markers with unknown position, stem rust responses and pedigrees of wheat lines in the 2^nd^ and 5^th^ stem rust resistance screening nurseries. (XLSX)
